# Modified Zeolite/Polysulfone Mixed Matrix Membrane for Enhanced CO_2_/CH_4_ Separation

**DOI:** 10.3390/membranes11080630

**Published:** 2021-08-16

**Authors:** Lanisha Devi Anbealagan, Tiffany Yit Siew Ng, Thiam Leng Chew, Yin Fong Yeong, Siew Chun Low, Yit Thai Ong, Chii-Dong Ho, Zeinab Abbas Jawad

**Affiliations:** 1 Department of Chemical Engineering, Faculty of Engineering, Universiti Teknologi PETRONAS, Seri Iskandar 32610, Perak, Malaysia; lanisha_19000945@utp.edu.my (L.D.A.); tiffany_921206@hotmail.com (T.Y.S.N.); yinfong.yeong@utp.edu.my (Y.F.Y.); 2 CO_2_ Research Center (CO_2_RES), Institute of Contaminant Management, Universiti Teknologi PETRONAS, Seri Iskandar 32610, Perak, Malaysia; 3 School of Chemical Engineering, Engineering Campus, Universiti Sains Malaysia, Nibong Tebal 14300, Pulau Pinang, Malaysia; chsclow@usm.my; 4 Department of Petrochemical Engineering, Faculty of Engineering and Green Technology, Universiti Tunku Abdul Rahman, Jalan Universiti, Bandar Barat, Kampar 31900, Perak, Malaysia; ongyt@utar.edu.my; 5 Department of Chemical and Materials Engineering, Tamkang University, New Taipei City 25137, Taiwan; cdho@mail.tku.edu.tw; 6 Department of Chemical Engineering, College of Engineering, Qatar University, Doha P.O. Box 2713, Qatar; zjawad@qu.edu.qa

**Keywords:** mixed matrix membranes (MMMs), polysulfone, zeolite RHO, silane modification, CO_2_ separation

## Abstract

In recent years, mixed matrix membranes (MMMs) have received worldwide attention for their potential to offer superior gas permeation and separation performance involving CO_2_ and CH_4_. However, fabricating defect-free MMMs still remains as a challenge where the incorporation of fillers into MMMs has usually led to some issues including formation of undesirable interfacial voids, which may jeopardize the gas separation performance of the MMMs. This current work investigated the incorporation of zeolite RHO and silane-modified zeolite RHO (NH_2_–RHO) into polysulfone (PSf) based MMMs with the primary aim of enhancing the membrane’s gas permeation and separation performance. The synthesized zeolite RHO, NH_2_–RHO, and fabricated membranes were characterized by X-ray diffraction (XRD) analysis, Fourier transform infrared-attenuated total reflection (FTIR-ATR), thermogravimetric analysis (TGA) and field emission scanning election microscopy (FESEM). The effects of zeolite loading in the MMMs on the CO_2_/CH_4_ separation performance were investigated. By incorporating 1 wt% of zeolite RHO into the MMMs, the CO_2_ permeability and ideal CO_2_/CH_4_ selectivity slightly increased by 4.2% and 2.7%, respectively, compared to that of a pristine PSf membrane. On the other hand, a significant enhancement of 45% in ideal CO_2_/CH_4_ selectivity was attained by MMMs incorporated with 2 wt% of zeolite NH_2_-RHO compared to a pristine PSf membrane. Besides, all MMMs incorporated with zeolite NH_2_-RHO displayed higher ideal CO_2_/CH_4_ selectivity than that of the MMMs incorporated with zeolite RHO. By incorporating 1–3 wt% zeolite NH_2_-RHO into PSf matrix, MMMs without interfacial voids were successfully fabricated. Consequently, significant enhancement in ideal CO_2_/CH_4_ selectivity was enabled by the incorporation of zeolite NH_2_–RHO into MMMs.

## 1. Introduction

Carbon dioxide (CO_2_) is commonly found in many industrial gas streams, such as natural gas stream and flue gas stream. CO_2_/CH_4_ and CO_2_/N_2_ separations are among the most important gas separation processes. This is because the presence of CO_2_ in the gas stream reduces the calorific value of the gas stream. In addition, CO_2_ makes the gas stream corrosive, which creates problems for the pipelines used for gas stream transportation. Membrane technology has received worldwide attention in the application of gas separation over the decades. This is due to the fact that membrane technology demonstrates advantages such as low energy consumption, compact design, simplicity of operation, flexibility of scale-up, possible use for continuous operation, and no requirement for phase change [1,2,3]. Polymeric membranes used to be very appealing in industrial gas separations due to its ease in scaling up and low fabrication cost. Nonetheless, these membranes commonly suffer an extremity in the tradeoff relation between permeability and selectivity [4]. Despite inorganic membranes offering advantages over polymeric membranes in terms of their high thermal stability, chemical resistance, and ability to offer relatively high gas permeability and selectivity, the application of inorganic membranes are restricted due to high fabrication cost and difficulty in fabricating defect-free membranes [5,6]. Hence, the development of mixed matrix membranes (MMMs) has been gaining increasing popularity among researchers in recent years.

As a promising new generation, MMMs are based on a system that combines two or more materials with excellent properties by embedding a dispersed inorganic filler into a continuous polymer matrix [1]. Relative to the current polymeric membranes, these MMMs offer a viable approach to achieve better gas permeability and selectivity, deriving from the incorporation of inorganic filler with inherent remarkable gas separation characteristics [7,8]. In this regard, zeolite filled MMMs appear to be a potential candidate for gas separation applications.

Zeolites are inorganic, microporous aluminosilicates that exhibit significant potential in gas separation due to their well-defined pore apertures and molecular sieving characteristics. The size selective characteristic of zeolite enables selective separation of smaller gas molecules from larger sized gas molecules [9,10]. Hence, this distinctive molecular sieving nature of zeolite can be favorable in enhancing the gas permeability and selectivity if it is incorporated into polymeric membranes. Zeolite RHO is receiving much attention for gas adsorption and separation as its framework has a 0.36 nm pore opening, which is in the vicinity of kinetic diameters of various gas molecules such as CO_2_ (0.33 nm) and CH_4_ (0.38 nm) [11]. In research reported by Atalay-Oral et al. [12], a high CO_2_ adsorption capacity of about 0.30 g/g was attained for zeolite RHO. In addition, the ratios of CO_2_ adsorption capacity to CH_4_ adsorption capacity as high as 74.9 was reported for zeolite RHO [12]. This indicates the potential of zeolite RHO to be used for CO_2_/CH_4_ separation.

In spite of the fact that zeolite filled MMMs display numerous advantages in gas separation, these membranes have been experiencing issues related to the formation of non-selective interfacial voids due to poor compatibility between polymer matrix and inorganic fillers, causing adverse impact on the separation performance [13]. In order to improve the compatibility of zeolite in polymer matrix, surface modification of zeolite with silane coupling agents on the surface of zeolite has been proposed to enhance the polymer/zeolite compatibility [14,15]. Silane coupling agents such as (γ-aminopropyl)-triethoxysilane (APTES), *N*-β-(aminoethyl)-γ-aminopropyltrimethoxysilane (APTMS), (γ-aminopropyl)-diethoxymethylsilane (APDEMS), and (γ-glycidyloxy-propyl)-trimethoxysilane (GLYMO) consist of two reactive groups, namely an organic functional group and an inorganic hydrolysable group [16,17,18,19]. R-(CH_2_)_n_-Si-X_4−n_ is the general formula of a silane coupling agent, wherein R is the organic functional group such as amine, methacryloxy, or epoxy group, whilst X acts as the methoxy, ethoxy, or acetoxy in hydrolysable group [14]. By hydrolyzing the coupling agents, free silanol groups (Si(OH)_4−n_) will be formed and will be able to react with hydroxyl groups on the external surface of zeolites via hydrogen bonding. The amine group acts as an active site and creates a hydrophobic bond at the interface between zeolite and polymer chain so as to promote adhesion between zeolite and polymer matrix [20,21,22]. In fact, the distinctive properties of silane coupling agents may not only alter the surface properties of zeolite from hydrophilic to hydrophobic, but also enhance zeolite affinity for the polymer matrix [23,24,25].

Amooghin et al. [15] investigated the effect of silane modification on Matrimid 5218/NaY MMMs with APDEMS for CO_2_/CH_4_ separation. It was clearly demonstrated that the polymer/zeolite interfacial adhesion was enhanced by silane-modified zeolite NaY particles and the particles were well distributed up to 15 wt% within the Matrimid matrix without any agglomeration. There was about a 16% increase in CO_2_ permeability from 8.34 Barrer for Matrimid membrane to 9.70 Barrer for MMMs incorporated with 15 wt% silanated NaY. The respective CO_2_/CH_4_ selectivity was also increased by approximately 57% from 36.3 to 57.1 [15]. Perchar et al. [26] used APTMS-modified ZSM-2 to prepare polyimide MMMs. Absence of voids between polymer and modified zeolite was revealed in SEM and TEM studies and modified zeolites were well distributed across the polymer surface [26]. In another work, Perchar et al. [27] further incorporated APTES-modified zeolite L into polyimide membranes. There was no interfacial voids between polyimide and modified zeolite L in observed SEM images, suggesting high affinity was established between polyimide and modified zeolite L [27]. In the preparation of polysulfone (PSf) MMMs, Junaidi et al. [14] modified SAPO-34 zeolite using APTMS in isopropanol and ethanol, respectively. Apart from reduction of filler agglomeration, it was proved that APTMS as silane coupling agent was able to reduce interfacial voids in MMMs. CO_2_ permeance of 706 GPU and CO_2_/CH_4_ selectivity of 31 were achieved by PSf/SAPO-34 MMMs with APTMS-modified SAPO-34 in ethanol [14]. Ismail et al. [28] fabricated polyethersulfone (PES) MMMs using APTES-modified zeolite 4A. Moreover, good compatibility between PES and silanated zeolite was noticed. A PES membrane incorporated with 20 wt% modified zeolite 4A yielded significant selectivity enhancement from 28.75 to 46.28 in CO_2_/CH_4_ separation.

To the best of our knowledge, there is no literature reported on the silane-modified zeolite RHO incorporated as filler to develop MMMs for CO_2_/CH_4_ separation. In this work, zeolite RHO was chosen as the filler in polysulfone (PSf) matrix to develop MMMs. CO_2_/CH_4_ permeation and separation studies were performed on the fabricated MMMs, because zeolite RHO has a 0.36 nm pore opening, which is between the kinetic diameter of CO_2_ (0.33 nm) and CH_4_ (0.38 nm) [11]. This work focused on an investigation of the effect of filler loading on the properties and CO_2_/CH_4_ separation performance of the MMMs. In addition, (3-aminopropyl)-triethoxysilane (APTES) was adopted as a silane coupling agent for surface modification of zeolite RHO. MMMs incorporated with APTES-modified zeolite RHO were also developed to investigate the enhancement in CO_2_/CH_4_ separation performance as well as in the properties of the MMMs. The obtained evaluation information from this work could serve as good indicator of the MMMs performance when the MMMs are to be applied in real-life industrial applications in gas processing.

## 2. Materials and Methods

### 2.1. Materials

Polysulfone (PSf) pellets (average M_n_ ~ 16,000, average M_w_ ~ 35,000, Sigma Aldrich, St. Louis, MO, USA) were used to fabricate pristine PSf membranes and their respective MMMs. Colloidal silica LUDOX HS-40 (40 wt%, Sigma Aldrich, St. Louis, MO, USA), cesium hydroxide (50 wt% aqueous solution, Sigma Aldrich), sodium aluminate (53 wt% Al_2_O_3_, 47 wt% Na_2_O, Sigma Aldrich, St. Louis, MO, USA), sodium hydroxide (>98%, Fisher Scientific, Hampton, NH, USA), and deionized water were used to synthesize zeolite RHO. (3-aminopropyl)-triethoxysilane (APTES) (99%) was acquired from Sigma Aldrich (St. Louis, MO, USA) and used without further purification. Tetrahydrofuran (THF) (>99.8%), toluene (>99.9%), and ethanol (>99.9%) were supplied by Merck Co (Kenilworth, NJ, USA). and employed without further purification. CO_2_ and CH_4_ gases (>99.5% purity) were provided by Air Products (Detroit, MI, USA).

### 2.2. Zeolite RHO Synthesis

Zeolite RHO was synthesized by following the method reported in our previous work [29]. A precursor solution with molar composition of 3 Na_2_O : 0.4 Cs_2_O : Al_2_O_3_ : 10.8 SiO_2_ : 110 H_2_O was prepared by dissolving sodium hydroxide in deionized water at room temperature. Then, cesium hydroxide and sodium aluminate were added to the mixture consecutively. After complete dissolution, colloidal silica was added to the solution under stirring for 30 min. The resultant precursor solution was further pretreated with 40 kHz of ultrasonic irradiation for 120 min in an ultrasonic bath (Sonica 2400 EP S3, Milan, Italy). The precursor solution was stirred for aging at room temperature for 24 h. The aged solution was then transferred into a stainless steel autoclave reactor for hydrothermal synthesis for 2 days at 100 °C. Upon completion of hydrothermal synthesis, the synthesized zeolite RHO were obtained via repeated centrifugation and rinsing with deionized water. The zeolite RHO was then dried in an oven at 80 °C overnight.

### 2.3. Silane Modification of Zeolite RHO

Zeolite RHO sample was dried overnight in oven at 80 °C before being used. The silane modification procedure was carried out by following Plueddemann’s method with modifications [25]. A total of 2 g of zeolite RHO powder was dispersed in 50 mL of toluene and stirred for one hour at room temperature. Then, 4 mL of APTES was added dropwise to the mixture and it was refluxed at 110 °C for 4 h. After 4 h, the mixture was left to cool to room temperature, followed by filtration and washing with toluene and ethanol to remove unreacted silane. The modified zeolite RHO was dried in the oven at 80 °C overnight.

### 2.4. Mixed Matrix Membrane Fabrication

PSf pellets, zeolite RHO, and silane-modified zeolite RHO (NH_2_-RHO) powder were pre-dried at 110 °C and 80 °C overnight, respectively, to remove moisture content. Priming method was used to fabricate the MMMs to minimize agglomeration of inorganic fillers [30]. Table 1 shows the sample names of pristine membranes and MMMs fabricated in the current study. Different loading of zeolite RHO and NH_2_-RHO, as described in Table 1, were added, to the THF solvent and ultrasonicated for 30 min to yield a homogenous particle dispersion. Then, priming was carried out by the addition of 10% of total amount of PSf pellets into the dope solution and stirred for 4 h to ensure PSf was effectively coated around zeolite particles [31]. The remaining PSf pellets were added in batches to form the final dope solution, which was further stirred overnight at room temperature. Prior to casting, the dope solution was degassed in an ultrasonic bath for 30 min to eliminate trapped micro-bubbles. The resultant dope solution was poured and cast on a cleaned flat levelled glass plate with a 0.2 mm gap of casting knife. The cast membrane layer on the glass plate was dried for 24 h at ambient pressure and temperature. Then, the membrane layer was detached from the glass plate and dried overnight in an oven at 75 °C to remove any residual solvent. For comparison purposes, a pristine PSf membrane was also fabricated by preparing a polymeric dope solution composed of 27 wt% PSf pellets in THF solvent. Lastly, the fabricated membranes were kept in a vacuum desiccator.

### 2.5. Zeolite and Membrane Samples Characterization

X-ray diffraction (XRD) analysis was conducted on the zeolite and membrane samples via diffractometer (X’Pert^3^ Powder & Empyrean, PANalytical, Malvern, UK) with monochromatic CuKα radiation at a wavelength of 0.154 nm, accelerating voltage of 40 kV and current of 40 mA. Fourier transform infrared-attenuated total reflection (FTIR-ATR) was performed using a spectrometer (Perkin Elmer, Frontier, Waltham, MA, USA) to identify the functional groups present in the zeolite and membrane samples. The cross-sectional images of the membrane samples were monitored by field emission scanning electron microscopy (FESEM) (Zeiss Supra 55VP, Jena, Germany). All membrane samples were fractured in liquid nitrogen and sputter-coated with gold. The samples were mounted on stainless-steel holders and observed at an accelerating voltage of 10 kV in high vacuum conditions. Thermogravimetric analysis (TGA) was employed to evaluate the thermal stability of the zeolite and membrane samples using an analyzer (Perkin Elmer, STA 6000, Waltham, MA, USA). The samples were subjected to heating at a heating rate of 10 °C/min under nitrogen atmosphere.

### 2.6. Gas Permeation and Separation Evaluation

**Figure 1:**
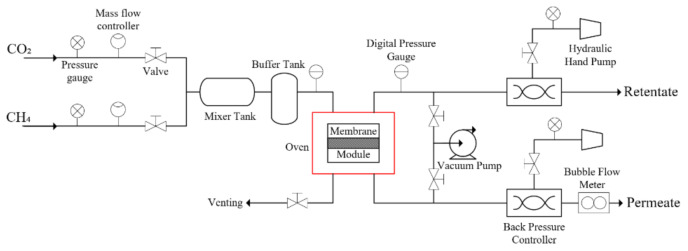
Schematic of the experimental setup used for gas permeation and separation studies.

The gas permeation and separation evaluation were carried out for pristine PSf membrane and MMMs with different filler loadings using the experimental setup as shown in Figure 1. The membrane sample was sealed in a flat sheet membrane module with a silicon gasket to prevent gas leakage. Single CO_2_ or CH_4_ gas was fed separately to the membrane sample in the membrane module. The gas permeation was conducted at 25 °C, where transmembrane pressure was regulated at 4 bar using back pressure regulator while the permeate pressure was kept at atmospheric pressure. Capillary bubble flowmeter was employed to measure the volumetric flow rate of the permeate. The CO_2_ and CH_4_ gas permeability were calculated with the following Equation (1):

(1) $$P=\frac{Q\;l}{A\;\Delta P}$$

where *P* is the gas permeability across the membrane in Barrer (1 Barrer = 1 × 10^−10^ cm^3^ (STP) cm/cm^2^ s cmHg), *Q* is gas flux (cm^3^ STP/s), *l* is membrane thickness (cm), *A* is effective membrane area (cm^2^) and Δ*P* is transmembrane pressure (cmHg) across the membrane. The ideal selectivity of the membrane was calculated as the ratio of CO_2_ permeability to CH_4_ permeability by using Equation (2):

(2) $$\alpha_{{\mathrm{CO}}_{2}/{\mathrm{CH}}_{4}}=\frac{P_{A}}{P_{B}}$$

where *P_A_* and *P_B_* are the CO_2_ and CH_4_ permeability, respectively. The gas permeation measurement of each single gas was repeated three successive times.

## 3. Results

### 3.1. Crystallographic Analysis

#### 3.1.1. Comparison of Zeolite RHO and NH_2_-RHO

**Figure 2:**
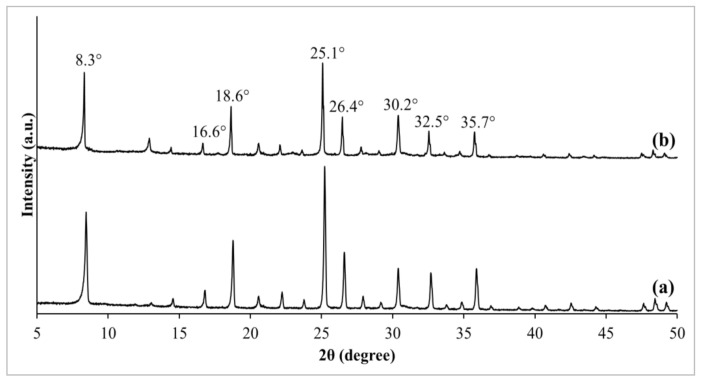
XRD patterns of (a) zeolite RHO and (b) zeolite NH_2_-RHO.

The crystallographic of zeolite RHO and NH_2_-RHO were ascertained by XRD analysis, as illustrated in Figure 2. As shown in Figure 2a, the synthesized zeolite RHO possessed characteristic diffraction peaks with respective 2θ angles at about 8.3°, 16.6°, 18.6°, 25.1°, 26.4°, 30.2°, 32.5°, and 35.7°, which is in agreement with the XRD patterns reported by Liu et al. [32]. According to Kim and Lee [33], sharp diffraction peaks with high intensity symbolize a highly crystalline region present in a synthesized sample. Zeolite NH_2_-RHO also portrayed similar sharp diffraction peaks to that of zeolite RHO as displayed in Figure 2b, which indicates a high degree of crystallographic regularity in the synthesized zeolite NH_2_-RHO. However, the characteristic peak intensity of zeolite NH_2_-RHO was slightly reduced compared to pure zeolite RHO. This situation can be explained by the incorporation of silane modification to the zeolite RHO surface where APTES slightly disrupted the crystalline structure of zeolite NH_2_-RHO, causing in a slight decrement of crystallinity [34].

#### 3.1.2. Comparison of Pristine PSf Membrane and MMMs

**Figure 3:**
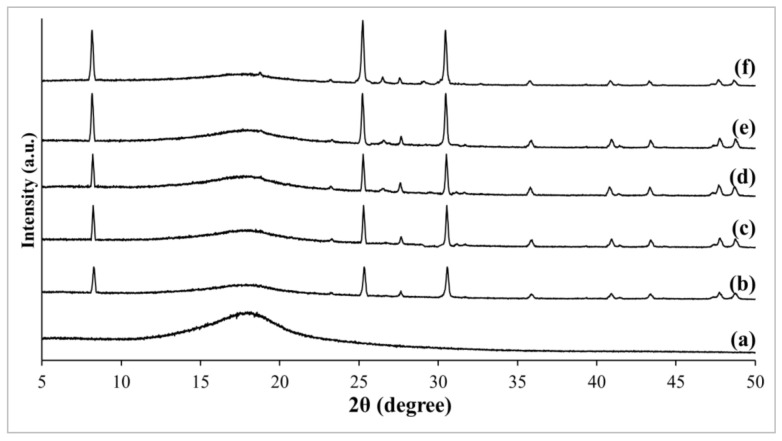
XRD patterns of (a) pristine PSf membrane, (b) 1RHO/PSf, (c) 2RHO/PSf, (d) 3RHO/PSf, (e) 4RHO/PSf, and (f) 5RHO/PSf MMMs.

**Figure 4:**
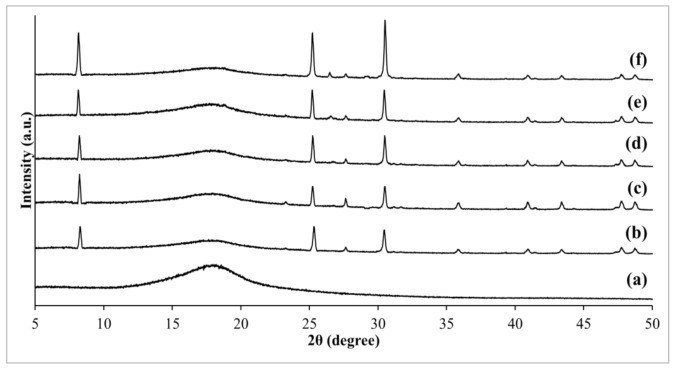
XRD patterns of (a) pristine PSf membrane, (b) 1NH_2_-RHO/PSf, (c) 2NH_2_-RHO/PSf, (d) 3NH_2_-RHO/PSf, (e) 4NH_2_-RHO/PSf, and (f) 5NH_2_-RHO/PSf MMMs.

The changes in crystallographic of pristine PSf membrane and fabricated MMMs were further analyzed and visualized in Figure 3 and Figure 4. Firstly, pristine PSf manifested amorphous polymer structure by revealing a broad, low intensity peak at a 2θ angle of about 18° [35]. Upon addition of zeolite RHO and NH_2_-RHO into PSf matrix, several additional sharp characteristic peaks with 2θ angles at about 8.3°, 25.1°, and 30.2° were observed in the MMMs, which indicates the presence of zeolite RHO and NH_2_-RHO in the MMMs. As the loading of zeolite RHO and NH_2_-RHO increased from 1 wt% to 5 wt%, the peak intensities at respective 2θ angles for zeolite RHO and NH_2_-RHO also increased. A similar phenomenon was reported by Ahmad et al. [36] when increasing peak intensity of zeolite 4A was observed in the Matrimid based MMMs as the loading of zeolite 4A increased from 10 wt% to 30 wt%.

### 3.2. Spectroscopic Analysis

#### 3.2.1. Comparison of Zeolite RHO and NH_2_-RHO

**Figure 5:**
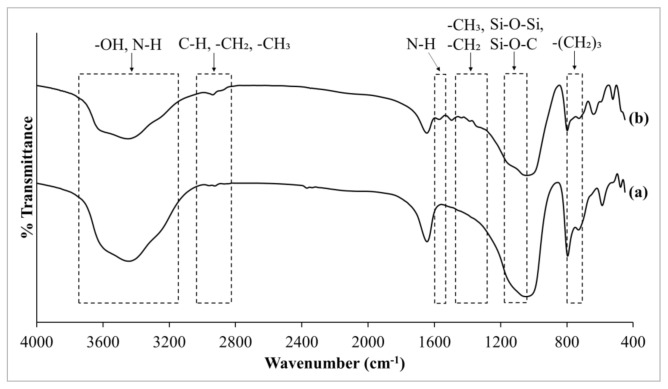
FTIR spectra of (a) zeolite RHO and (b) zeolite NH_2_–RHO.

Figure 5 depicts the FTIR spectra of zeolite RHO and NH_2_-RHO. The observed FTIR spectra differences between zeolite RHO and NH_2_-RHO are also indicated in Figure 5. As can be seen in Figure 5a for zeolite RHO, the FTIR spectrum portrayed a strong absorption peak in the range of 1250–950 cm^−1^, which corresponds to Si–O and Al–O tetrahedra asymmetrical stretching vibration in the zeolite RHO particle [29,37]. Intense absorption peaks located in the range of 3650–3200 cm^−1^ and 1640 cm^−1^ indicated the presence of O–H stretching of adsorbed water molecules and O–H bending of lattice water in the zeolite RHO sample [38]. Besides, a low intensity absorption peak identified at 794 cm^−1^ can be connected to Si–Al–O symmetrical stretching vibration. The characteristic absorption peak of double eight-ring external linkage in zeolite RHO was revealed at 588 cm^−1^ and 634 cm^−1^ [29,38].

As observed from Figure 5b for NH_2_–RHO, the absorption peak ranging from 3650–3200 cm^−1^ showed an increasing broadness compared to that of zeolite RHO. This can be ascribed to the presence of N-H stretching vibration of primary amine from APTES, which was overlapping with O–H stretching vibrations of hydroxyl group in the same frequency spectra range [34]. Moreover, a slight increase at the absorption peak of about 2937 cm^−1^ was attributed to aliphatic C–H, –CH_2_, –CH_3_ stretching vibrations due to the introduction of APTES onto zeolite RHO [28]. This is in agreement with Ismail et al. [28] where APTES was used to modify the surface of zeolite 4A. The zeolite NH_2_–RHO also displayed the increased intensity of absorption peaks within the range of 1500–1300 cm^−1^, which represents the Si–CH_2_- and Si–CH_3_ stretching vibrations of silane groups. Likewise, the observed increment in broadness of absorption peaks ranging from 780–726 cm^−1^ for zeolite NH_2_-RHO was connected to –(CH_2_)_3_– rotations and vibrations [14]. These proved that the silane coupling agent, APTES was adhered to the surface of zeolite RHO. An analogous observation was reported by Amooghin et al. [15] in the surface modification of zeolite NaY by APDEMS.

According to Ismail et al. [28], the physical and chemical adsorption reactions of silane coupling agents can be further verified by the silanol groups on the zeolite surface. There is a weak, broad overlapped absorption peak at about 1097 cm^−1^, related to asymmetric stretching of Si–O–Si with Si–O–C stretching vibration, which originates from non-bridging O–H stretching vibration. Hydrogen bonding was created between the O atom of an OH group bonded to the Si atom of zeolite RHO surface and H atom of an OH group bonded to the Si atom of the APTES molecule [28]. In addition, the N-H bending vibration of primary amine was also noticeable in Figure 5b with the additional absorption peak at the frequency of about 1562 cm^−1^ for zeolite NH_2_–RHO, but it was absent in the spectrum of zeolite RHO (Figure 5a) [34]. In other words, the silane modification on zeolite RHO surface had successfully taken place.

#### 3.2.2. Comparison of Pristine PSf Membrane and MMMs

**Figure 6:**
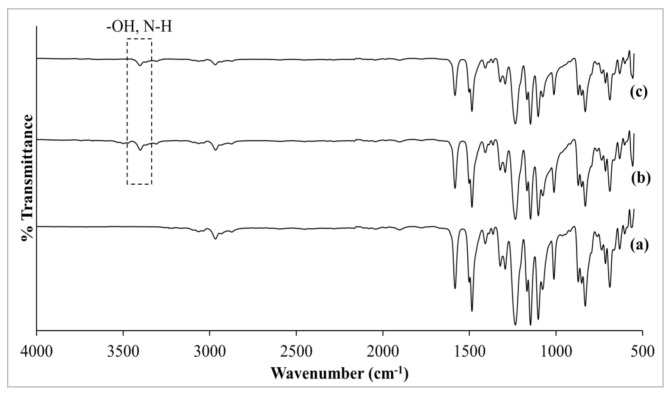
FTIR spectra of (a) pristine PSf membrane, (b) 5RHO/PSf, and (c) 5NH_2_-RHO/PSf MMMs.

Figure 6 shows the FTIR-ATR spectra of pristine PSf membrane, RHO/PSf and NH_2_-RHO/PSf MMMs. Based on Figure 6a, FTIR spectrum of pristine PSf membrane exhibited characteristic absorption peaks at about 1295 cm^−1^ and 1323 cm^−1^, corresponding to the presence of O=S=O stretching vibration. The absorption peaks of C–H stretching and bending vibrations of aliphatic rings were identified at about 2967 cm^−1^ and 1364 cm^−1^, respectively. –(CH_2_)_3_– rotations and vibrations were also visible at the absorption peaks ranging from 3070–2850 cm^−1^ and 770–630 cm^−1^, respectively [14,39]. In the meantime, stretching vibration of C=C due to benzene ring appeared as a medium intensity absorption peak at about 1485 cm^−1^ and 1584 cm^−1^ [14,15,40]. There is a strong intensity absorption peak at about 1235 cm^−1^, which was ascribed to C–O stretching vibration where the oxygen atom is bonded to two phenyl groups. An analogous observation was also reported by Muntha et al. [40] in the fabrication of PSf/zeolite 3A MMMs. 

The fabricated MMMs with zeolite RHO and NH_2_–RHO portrayed similar and comparable FTIR spectra to pristine PSf membranes, as displayed in Figure 6b,c. Intense absorption peaks in the range of 1080–960 cm^−1^ were attributed to the asymmetrical stretching vibration of Si–O and Al–O tetrahedral in the zeolite RHO and NH_2_–RHO [41]. However, both RHO/PSf and NH_2_-RHO/PSf MMMs revealed higher shoulder band within the range of 1075–1020 cm^−1^ compared with pristine PSf membranes. Furthermore, an overlapped peak at about 1012 cm^−1^ was represented by Si–O–C stretching vibration [14]. A similar observation was reported by Junaidi et al. [14] where a SAPO–34 zeolite was incorporated into a PSf matrix. 

It is evident that the characteristic sulfone peaks were maintained but the peak intensities slightly decreased by incorporating zeolite RHO and NH_2_-RHO into the PSf matrix. On the other hand, an additional absorption peak at about 3402 cm^−1^ for N–H stretching vibration of primary amine was observed in Figure 6, indicating the presence of APTES on the zeolite surface. The relative intensities were weakened due to superimposing frequency spectra of O–H stretching vibration of zeolite RHO in the range of 3600–3000 cm^−1^ [34]. 

### 3.3. Thermal Analysis

#### 3.3.1. Comparison of Zeolite RHO and NH_2_-RHO

**Figure 7:**
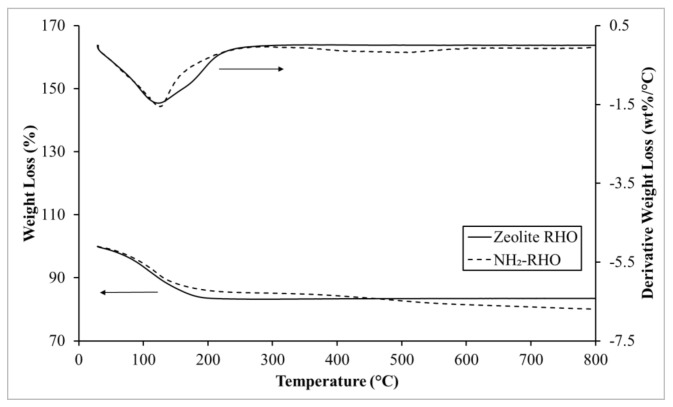
TGA-DTG thermograms of zeolite RHO and NH_2_-RHO.

Figure 7 illustrates the TGA-DTG thermogram for zeolite RHO and NH_2_–RHO. In fact, the synthesized zeolite RHO and NH_2_-RHO possessed high thermal stability upon heating from 30 to 800 °C. As visualized in Figure 7, both zeolite RHO and NH_2_-RHO manifested a sharp weight loss from about 30 to 250 °C ascribing to the desorption and removal of physically adsorbed water within zeolite RHO pores [42]. At 800 °C, the total weight loss for zeolite NH_2_-RHO was 29.9%, as compared to 16.5% weight loss for zeolite RHO. This situation can be attributed to the additional decomposition of silane coupling agent modified on the surface of zeolite RHO, which took place from about 350 to 550 °C [43].

#### 3.3.2. Comparison of Pristine PSf Membrane and MMMs

**Figure 8:**
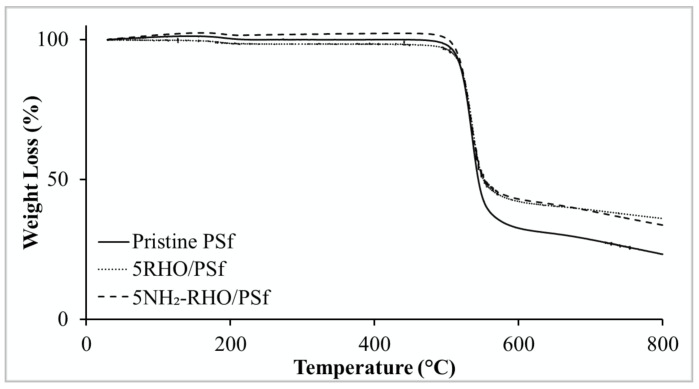
TGA thermograms of pristine PSf membrane, 5RHO/PSf, and 5NH_2_-RHO/PSf MMMs.

**Figure 9:**
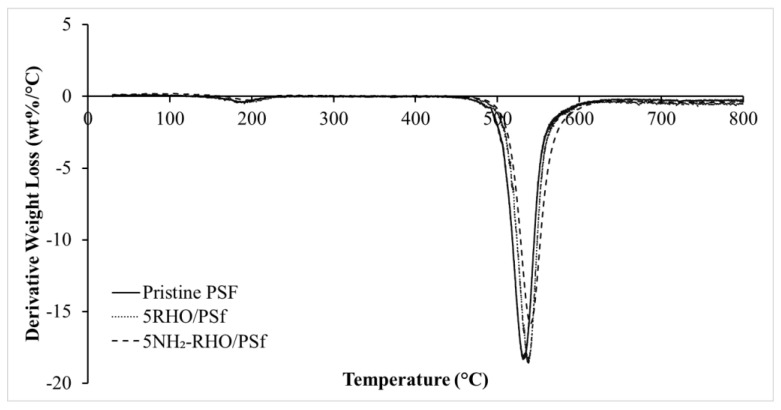
DTG thermograms of pristine PSf membrane, 5RHO/PSf, and 5NH_2_-RHO/PSf MMMs.

Thermal stability of pristine PSf membrane and MMMs were investigated via TGA-DTG analysis, and the results are shown in Figure 8 and Figure 9 while Table 2 depicts the decomposition temperature of the membranes. Referring to the TGA and DTG thermograms in Figure 8 and Figure 9, several observations could be drawn from the incorporation of zeolite RHO and NH_2_–RHO in PSf polymer matrix. Firstly, the membranes experienced two obvious stages of weight loss, where the first stage occurred at about 30 to 250 °C, indicating desorption of moisture and solvent residue during fabrication [44]. Subsequently, the second stage occurred above 460 °C, which ascribed to thermal decomposition of the main polymer chain [15]. The decomposition temperature, *T_d_* for pristine PSf was observed at 532.86 °C and it shifted slightly to higher values for the MMMs incorporated with 5 wt% of zeolite RHO or NH_2_–RHO, as illustrated in Figure 9.

In order to explore the effect of zeolite on the thermal stability of the PSf matrix, different loading of zeolite RHO or NH_2_-RHO were added to the PSf matrix. It was observed from Table 2 where the *T_d_* trend of MMMs increased gradually with increasing loading of zeolite RHO or NH_2_–RHO. This indicates that the thermal stability of MMMs was enhanced gradually with increasing loading of zeolite RHO or NH_2_–RHO in the MMMs [45]. Generally, increasing zeolite filler loading in the polymer matrix would enhance the interaction between polymer and filler. Strong hydrogen bond and covalent bond formed between zeolite RHO or NH_2_–RHO with PSf matrix confine the thermal motion of polymer chain and hence, higher energy would be required for the segmentation and decomposition of polymer chains [46].

### 3.4. Morphological Analysis

**Figure 10:**
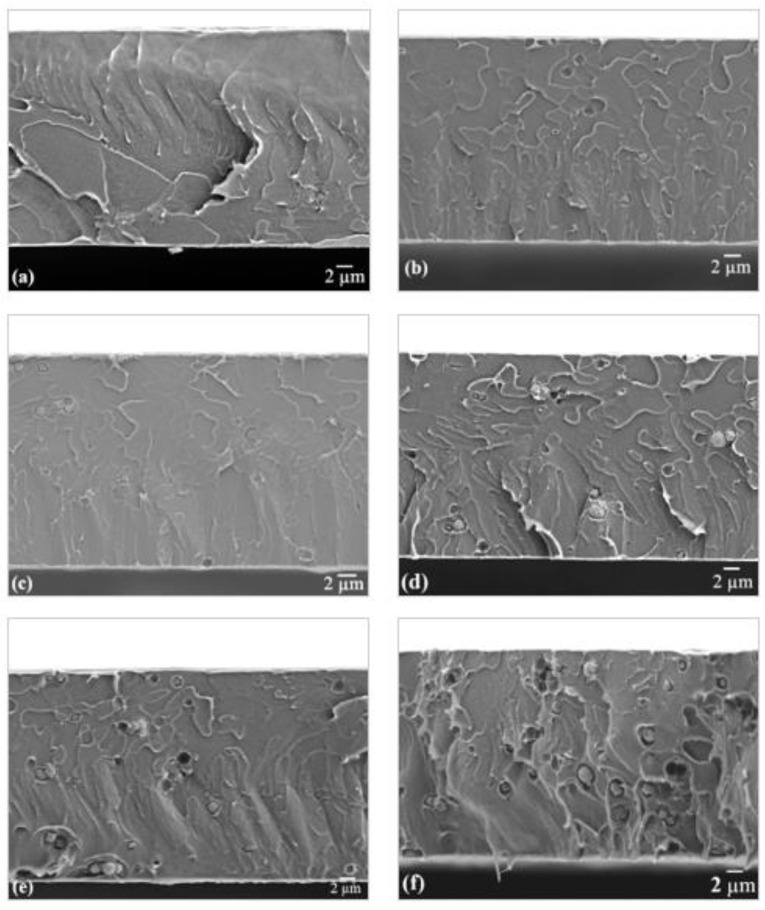
Cross sectional FESEM images of (**a**) pristine PSf membrane, (**b**) 1RHO/PSf, (**c**) 2RHO/PSf, (**d**) 3RHO/PSf, (**e**) 4RHO/PSf, and (**f**) 5RHO/PSf MMMs.

FESEM was performed to provide a visualization of the membrane structure and morphology. Figure 10 portrays the cross-sectional FESEM image of the pristine PSf membrane and the MMMs incorporated with zeolite RHO. Upon incorporation of 1 to 5 wt% of zeolite RHO particles into PSf polymer matrix, the appearance of zeolite RHO particles across PSf polymer matrix were observed. There were few empty cavities representing zeolite RHO particles being cleaved away when the MMMs were fractured with liquid nitrogen. It is observed that the membrane morphology was smoother when low loading of zeolite RHO was incorporated into the membrane. However, the formation of interfacial voids was detected between zeolite RHO particles and polymer matrix in the MMMs incorporated with higher loading of zeolite RHO as shown in Figure 10, which might be due to partial incompatibility between PSf polymer matrix and zeolite RHO [47]. The effect of partial incompatibility between PSf polymer matrix and zeolite RHO was greater at higher loading of zeolite RHO. An analogous phenomenon was reported by Huang et al. [47] when zeolite beta was incorporated into PSf matrix. As zeolite RHO loading increased beyond 4 wt%, obvious zeolite RHO particle agglomeration and severe interfacial voids at PSf/zeolite RHO interfacial region can be observed in Figure 10. Similarly, the formation of interfacial voids and filler agglomeration, especially at high zeolite loadings, has also been reported for several zeolite-filled MMMs by Amooghin et al. [46], Huang et al. [47], and Li et al. [48].

**Figure 11:**
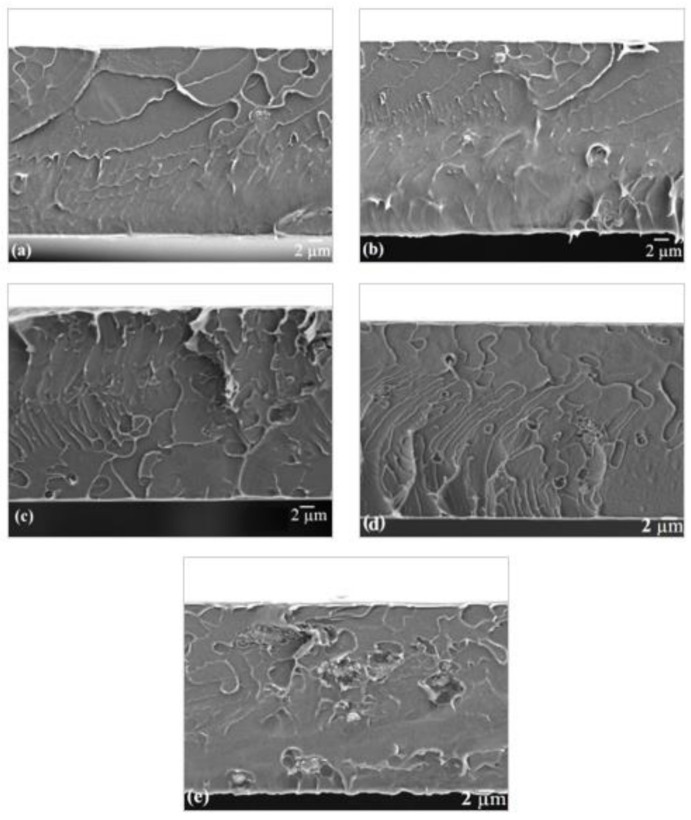
Cross sectional FESEM images of (**a**) 1NH_2_-RHO/PSf, (**b**) 2NH_2_-RHO/PSf, (**c**) 3NH_2_-RHO/PSf, (**d**) 4NH_2_-RHO/PSf, and (**e**) 5NH_2_-RHO/PSf MMMs.

Cross sectional FESEM images of the fabricated MMMs with different NH_2_-RHO loadings were illustrated in Figure 11. Upon addition of 1 to 3 wt% of NH_2_-RHO particles to the MMMs, no obvious interfacial voids between NH_2_-RHO particles and PSf matrix can be observed. In the presence of APTES, interfacial strength between PSf and NH_2_-RHO was enhanced significantly, resulting in stronger polymer/inorganic filler adhesion. The silane groups of APTES are able to bind with the hydroxyl groups on the zeolite RHO surface, while the amino group can bind with the sulfone group in PSf, forming hydrogen and covalent bonds between PSf and NH_2_-RHO particles [20]. Similar observations were found by Amooghin et al. [15] for APDEMS-modified zeolites with Matrimid.

### 3.5. Gas Permeation and Separation Evalution

#### 3.5.1. Effect of Zeolite RHO Loadings

**Figure 12:**
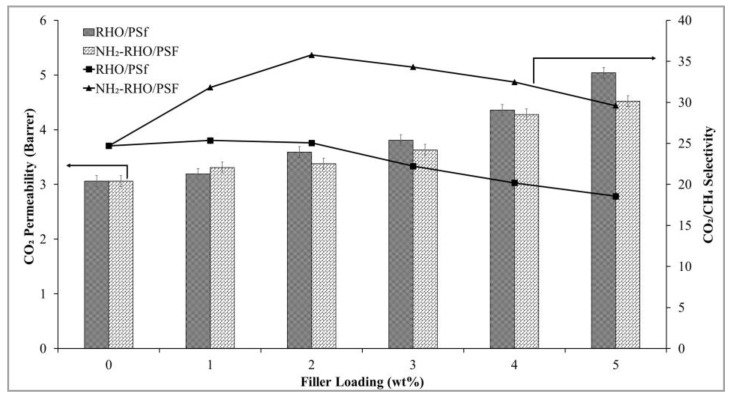
Gas permeation and separation performance of pristine PSf membrane and MMMs.

Figure 12 displays the gas permeation and separation performance of pristine PSf membrane and RHO/PSf MMMs. CO_2_ permeability and ideal CO_2_/CH_4_ selectivity of pristine PSf membrane in this study were comparable with those reported in the literature [49,50]. When 1 wt% of zeolite RHO was incorporated into PSf based MMMs, the CO_2_ permeability and ideal CO_2_/CH_4_ selectivity were slightly increased by 4.2% and 2.7%, respectively, compared to that of the pristine PSf membrane. The slight enhancement of ideal CO_2_/CH_4_ selectivity could be contributed by the molecular sieving effect of small pore zeolite RHO incorporated into the MMMs. CO_2_ gas molecules with smaller kinetic diameter could permeate through RHO/PSf MMMs with less resistance compared to CH_4_ gas molecules [11,12]. In addition, the polar character of zeolite RHO has greater affinity towards CO_2_ [51], leading to the slight improvement in ideal CO_2_/CH_4_ selectivity when 1 wt% of zeolite RHO was incorporated into PSf based MMMs in the current study.

However, increasing zeolite RHO loading beyond 1 wt% has resulted in the increase in CO_2_ permeability and increase in CH_4_ permeability but decrease in the ideal CO_2_/CH_4_ selectivity. This might be due to weak interaction between PSf matrix and zeolite RHO, the effect of which was greater at higher zeolite loading levels. Incorporation of low loading of 1 wt% zeolite RHO into PSf did not obviously affect the membrane morphology. When the zeolite RHO loading was higher than 1 wt%, the effect of weak interactions between the PSf matrix and zeolite RHO started to take place and the interfacial voids started to form at the PSf/zeolite RHO interface, plausibly produced a gaseous bypass between PSf polymer chain and incorporated zeolite RHO particles [47]. The free volume between polymer chain and zeolite RHO increased, causing a leaky interface with the presence of non-selective interfacial voids at the PSf/zeolite RHO interfacial region [27]. These interfacial voids are non-selective as it allows gas molecules to pass through the voids with less resistance instead of passing through zeolite pores [52]. Hence, the CO_2_ permeability increased but the ideal CO_2_/CH_4_ selectivity was sacrificed when zeolite RHO loading in the MMMs increased from 1 to 5 wt%.

#### 3.5.2. Effect of Zeolite NH_2_-RHO

Gas permeation and separation performance of NH_2_-RHO/PSf MMMs is depicted in Figure 12. Based on Figure 12, CO_2_ permeability of NH_2_-RHO/PSf MMMs showed an increasing trend with increasing NH_2_-RHO loading in the MMMs. A significant increase of 45% in ideal CO_2_/CH_4_ selectivity was achieved when 2 wt% of NH_2_-RHO was incorporated into the MMMs compared to the pristine PSf membrane. Besides, NH_2_-RHO/PSf MMMs with 2 wt% of NH_2_-RHO content exhibited about 43% higher ideal CO_2_/CH_4_ selectivity compared to that of RHO/PSf MMMs with 2 wt% of zeolite RHO loading. The enhanced ideal CO_2_/CH_4_ selectivity of NH_2_-RHO/PSf MMMs was due to the molecular sieving effect by the small pore zeolite NH_2_-RHO incorporated into the MMMs. In addition, APTES modified on zeolite RHO strengthened the interfacial interaction between PSf and NH_2_-RHO particles as well as significantly reduced the formation of interfacial voids, producing MMMs with enhanced morphology [22]. Therefore, the increase in ideal CO_2_/CH_4_ selectivity with the incorporation of 2 wt% of NH_2_-RHO into the MMMs was contributed by the small pore size of NH_2_-RHO, as well as enhanced MMMs morphology. These results were in line with FESEM observations as shown in Figure 11 where no obvious interfacial void can be seen at the PSf/NH_2_-RHO interface for incorporation of NH_2_-RHO particles of up to 3 wt% loading.

## 4. Conclusions

MMMs incorporated with zeolite RHO and NH_2_-RHO particles have been successfully fabricated in the current study. The incorporation of NH_2_-RHO particles has contributed to the enhanced thermal stability of MMMs as indicated in the TGA-DTG analysis. Furthermore, by incorporating 1–3 wt% zeolite NH_2_-RHO into the PSf matrix, MMMs without interfacial voids were successfully fabricated due to the enhancement in polymer/filler interfacial strength. Consequently, the NH_2_-RHO/PSf MMMs displayed higher ideal CO_2_/CH_4_ selectivity compared to RHO/PSf MMMs. A significant increase of 45% in ideal CO_2_/CH_4_ selectivity was achieved when 2 wt% of NH_2_-RHO was incorporated into the MMMs compared to pristine PSf membrane. The silane coupling agent (APTES) played an important role in strengthening the interfacial interaction between PSf and NH_2_-RHO particles and thereby significantly enhanced the ideal CO_2_/CH_4_ selectivity of the MMMs. The results obtained in this work contribute to the advancement of knowledge of the fabrication of membranes. Future research could be focused on investigation of the membranes in other configurations with higher membrane surface areas.

## Figures and Tables

**Table 1:** The pristine membrane and MMMs fabricated in current study.

Filler Loading in PSf (wt%)	MMMs with Zeolite RHO	MMMs with Silane-Modified Zeolite RHO
0	Pristine PSf	Pristine PSf
1	1RHO/PSf	1NH_2_-RHO/PSf
2	2RHO/PSf	2NH_2_-RHO/PSf
3	3RHO/PSf	3NH_2_-RHO/PSf
4	4RHO/PSf	4NH_2_-RHO/PSf
5	5RHO/PSf	5NH_2_-RHO/PSf

**Table 2:** Decomposition temperature of pristine PSf membrane and MMMs.

Sample	Decomposition Temperature, *T_d_* (°C)
Pristine PSf	532.86
1RHO/PSf	536.46
2RHO/PSf	537.02
3RHO/PSf	537.22
4RHO/PSf	537.63
5RHO/PSf	538.22
1NH_2_-RHO/PSf	539.16
2NH_2_-RHO/PSf	539.29
3NH_2_-RHO/PSf	539.87
4NH_2_-RHO/PSf	540.53
5NH_2_-RHO/PSf	540.70

## Data Availability

Not applicable.
